# The complete chloroplast genome of *Viola philippica*

**DOI:** 10.1080/23802359.2021.1906176

**Published:** 2021-04-22

**Authors:** Yupeng Guo, Pengcheng Lin, Min Wang

**Affiliations:** a College of Ecological Environment and Resources, Qinghai Nationalities University, Xining, PR China; b College of Pharmacy, Key Laboratory for Qinghai-Tibet Plateau Phytochemistry of Qinghai Province, Qinghai Nationalities University, Xining, PR China

**Keywords:** *Viola philippica*, chloroplast genome, phylogenetic analysis

## Abstract

The complete chloroplast genome of *Viola philippica* was sequenced, assembled, and annotated. It is a circular form of 156,469 bp in length, which was separated into four distinct regions, a large single-copy (LSC) of 85,668 bp, a small single-copy region (SSC) of 18,001 bp, and two inverted repeats (IR) of 26,400 bp. After annotation, a total of 129 genes were predicted, of which, 84 encode proteins, 8 rRNA, and 37 tRNA. The evolutionary history, inferred using maximum likelihood (ML) method, indicates that *V. philippica* was grouped within Violaceae, and comprised a clade with *Viola seoulensis* with 100% Bootstrap value.

*Viola philippica,* belonging to Violaceae, is a widely distributed perennial herb in China (Zhang et al. 2014). For containing bioactive constituents, such as Lignans, flavonoids, and coumarins, it was used extensively in traditional Chinese medicines for heat-clearing, detoxification, anti-inflammation, and pain relief (He et al. 2011; Wang et al. 2019; Yu et al. 2019). In previous studies, although many literatures on chemical composition and their functions have been documented, only a few literatures were about its genetic research (Zhang et al. 2014; Li et al. 2016). In this study, we report the complete chloroplast (cp) genome of *V. philippica*.

Samples from Qilian mountains (36°35′18″N, 101°49′33″E) in Qinghai province were collected for sequencing. Voucher specimen (HCPQNU-20200602001) was deposited in the Herbarium, College of Pharmacy, Qinghai Nationalities University. A sample’s total genomic DNA was extracted from about 100 mg fresh leaves using a modified CTAB method (Murray and Thompson 1980). Paired-end Libraries with an average length of 350 bp were constructed and sequenced on Illumina Novaseq 6000 platform (Shenzhen Huitong Biotechnology Co. Ltd, Shenzhen, China). The complete cp genome was assembled *via* the *de novo* assembler SPAdes (Bankevich et al. 2012) and annotated *via* PGA (Qu et al. 2019) with *Viola seoulensis* (KP749924) chloroplast genome as reference genome.

The complete cp genome of *V. philippica* (GenBank accession no. MT796627.1) has a typical quadripartite form of 156,469 bp in length, and composed of a large single-copy region (LSC, 85,668 bp), a small single-copy region (SSC, 18,001 bp), and two inverted repeats (IR, 26,400 bp). GC content of the genome is 36.3%. A total of 129 genes were predicted on this cp genome, of which, 84 encode proteins, 8 rRNA, and 37 tRNA.

Phylogenetic analysis was performed based on complete cp genomes of *V. philippica* and other seven related species reported in Violaceae, three species in Theaceae as out-group. The sequences were aligned using HomBlocks (Bi et al. 2018). The evolutionary history was inferred using maximum likelihood (ML) method in MEGA X (Kumar et al. 2018) with general time-reversible nucleotide substitution, Gamma distributed (GTR + G) model, and partial deletion of gaps/missing data. Bootstrap (BS) values were calculated from 1000 replicate analysis (Figure 1). As expected, *V. philippica* was grouped within Violaceae, and comprised a clade with *Viola seoulensis* with 100% BS value. The complete cp genome of *V. philippica* will be helpful for further studies on population genetics, taxonomy, or resource protection.

**Figure 1.**
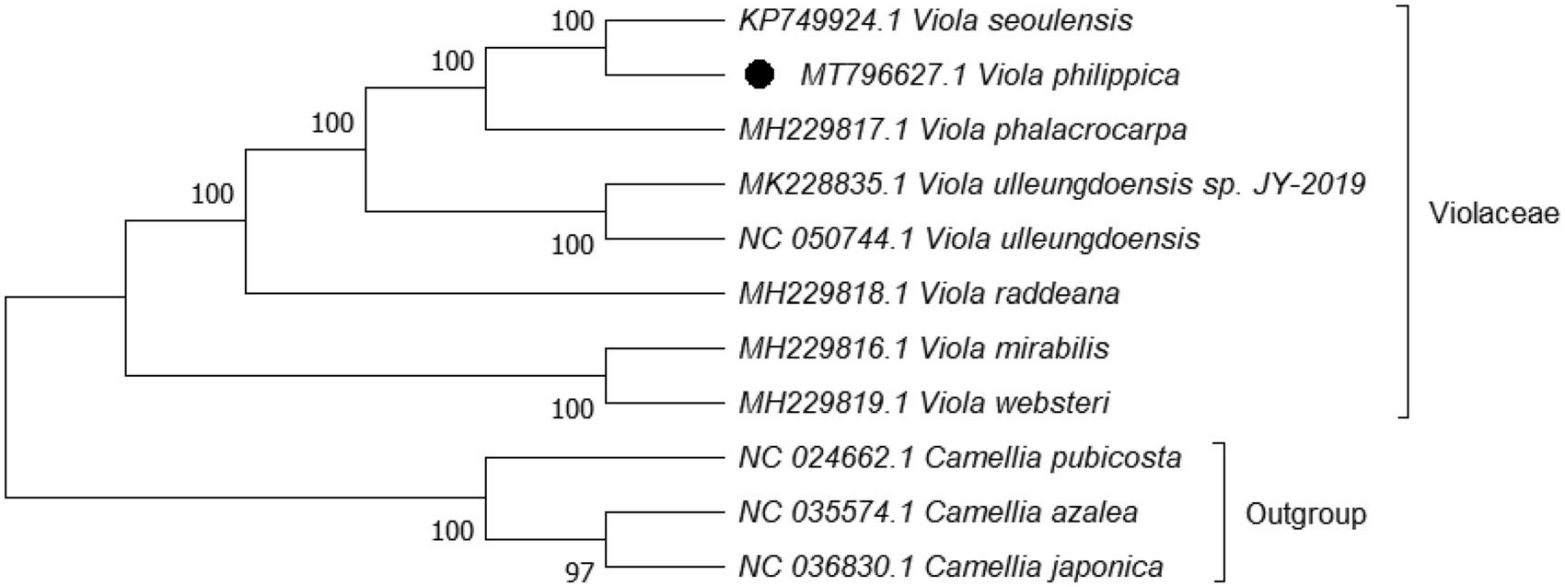
ML phylogenetic tree based on 11 species chloroplast genomes was constructed using MEGA X. Numbers on each node are bootstrap from 1000 replicate.

## Data Availability

The genome sequence data that support the findings of this study are openly available in GenBank of NCBI at (https://www.ncbi.nlm.nih.gov/nuccore/MT796627.1) under the accession no. MT796627.1. The associated BioProject, SRA, and Bio-Sample numbers are PRJNA678799, SRR13070133, and SAMN16812861, respectively.
